# The *Drosophila* Genome: So That's What it Looks Like!

**DOI:** 10.1002/1097-0061(20000630)17:2<154::AID-YEA18>3.0.CO;2-Z

**Published:** 2000

**Authors:** Rachel Drysdale, Leyla Bayraktaroglu

**Affiliations:** 1 FlyBase—Cambridge Department of Genetics University of Cambridge Downing Street Cambridge CB2 3EH UK; 2 FlyBase—Harvard, Department of Molecular and Cellular Biology Harvard University 16 Divinity Ave Cambridge MA 02138 USA


**Yeast**
*Yeast* 2000; **17**: 154–157.

**Meeting Review**

### The *Drosophila* genome: so that's what it looks like!

### 41st Annual *Drosophila* Research Conference, Lawrence Convention Center, Pittsburgh, USA. 22–26 March, 2000. Program Chairs: Pamela K. Geyer and Lori L. Wallrath. Sponsors: The Genetics Society of America

Rachel Drysdale[1]* and Leyla Bayraktaroglu[2]

[1] *FlyBase—Cambridge, Department of Genetics, University of Cambridge, Downing Street, Cambridge, CB2 3EH, UK*
[2] *FlyBase—Harvard, Department of Molecular and Cellular Biology, Harvard University, 16 Divinity Ave, Cambridge, MA, 02138, USA.*

**Correspondence to:*
*R. Drysdale, FlyBase—Cambridge,*
*Department of Genetics,*
*University of Cambridge, Downing Street, Cambridge, CB2 3EH, UK.*
*E-mail: r.drysdale@gen.cam.ac.uk*

When the participants in the 41st Annual *Drosophila* Research Conference returned to their labs after the meeting, their working landscape had changed. The annotated sequence of the euchromatic *Drosophila melanogaster* genome had become public information as a result of the unprecedented collaboration between the private Celera Genomics company and the public *Drosophila* Genome Project consortium (Berkeley *Drosophila* Genome Project, BDGP [8]; European *Drosophila* Genome Project, EDGP [4]; Baylor College of Medicine Human Genome Center [9]; FlyBase [5]). The afternoon of 23 March saw more than 1200 conference participants gathered for a landmark Genome Workshop that discussed the sequencing and annotation projects.

### The Genome Workshop

Gary Karpen, President of the *Drosophila* Board, introduced the Genome Workshop. He acknowledged the many groups that have participated in the genome sequencing collaboration. The success of this project has been based on contributions ranging from the mapping, cloning and sequencing of individual genes by researchers, through the publicly funded genome projects such as the BDGP, to the large-scale corporate investment by Celera Genomics. Gary led a standing ovation in recognition of all these contributions, and to acknowledge the debt owed to *Drosophila* researchers all the way back to the Morgan Fly Room. This lent a delightful sense of celebration to the afternoon.

Craig Venter (President, Celera Genomics, who has himself previously contributed to the *Drosophila* literature [17]) recounted how he had become interested in applying whole genome shotgun sequencing to *Drosophila* and introduced two talks in the **'Whole Genome Shotgun Sequencing'** section. Mark Adams (Celera Genomics) described the process whereby the genome sequence was obtained using three libraries with 2 kb, 10 kb and 130 kb inserts and a lot of sequencing machines. Mark then summarized how the gene predictions were generated, analysed with respect to encoded function by comparison to known proteins of other organisms, and correlated with the FlyBase-compiled set of 2783 gene coding sequences previously known to have substantial sequence information (see [1] for full description). Gene Myers (Celera Genomics) then described the strategies applied to the computational analysis, whereby individual sequence runs are assembled, using a 'paired-end' strategy, into scaffolds which include blocks of contiguous sequence (see [12] for a full description).

Gerry Rubin (Director, BDGP) then spoke on **'Comparative Genomics and the cDNA Project'** (see

Copyright © 2000 John Wiley & Sons, Ltd.

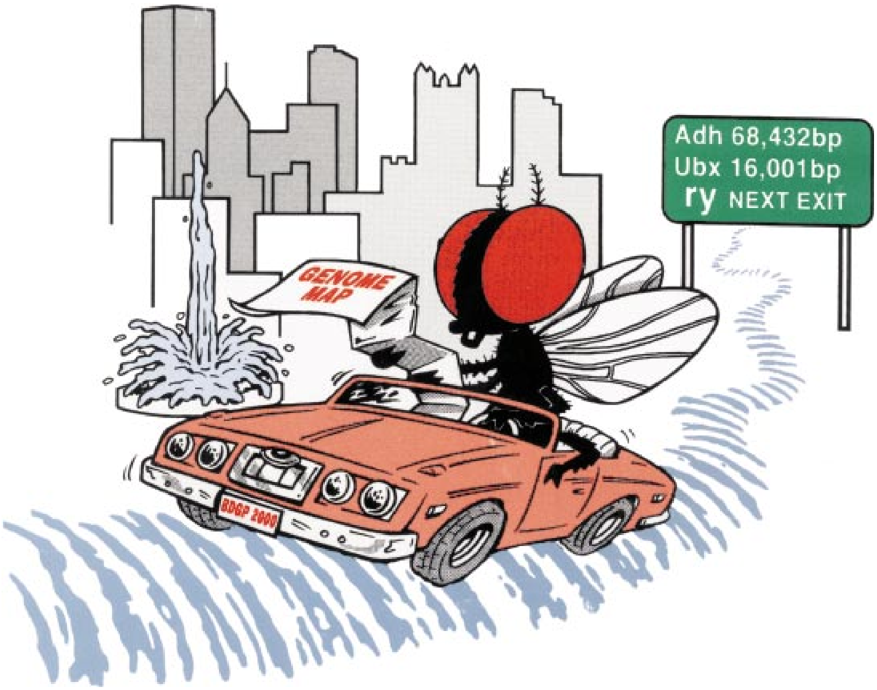

also [14,15]). Those for whom their primary interest in *Drosophila* is as a model organism will be struck by the observation that of 289 genes known to be altered in human diseases, 177 have orthologues in the fly genome, several of which, e.g. the orthologue of MEN (multiple endocrine neoplasia type 1), were previously unidentified [14]. The similarities and differences between the apportionment of protein families in the *D. melanogaster* and *Caenorhabditis elegans* genomes will occupy biologists for hours of happy puzzling. Of particular interest with respect to the progression from this 'Release 1.0' genome annotation to the next, more mature, version, Gerry described his project to generate the '*Drosophila* Gene Collection'. This unigene set of full-length cDNAs corresponding to 42% of predicted genes is due to be completed by June of this year [15].

Susan Celniker (Co-director of Sequencing, BDGP) spoke about **'Finishing the Genome Sequence'** and explained the process of filling the remaining sequence gaps within the scaffolds and bringing the sequence quality to a uniformly high standard of accuracy (Phase 3 standard; The published sequence is 99.99% accurate in areas of high quality but accuracy drops to 99.5% in areas with repetitive sequences). The mapped scaffolds of the sequence assembly included 1630 sequence gaps. These are now being filled by directed sequencing. The average size of the gaps filled in the first stage of gap filling was 771 bp (had been predicted to be 757 bp) and the predicted size of the remaining gaps is 2120 bp [1,12].

To close the Genome Workshop, Suzanna Lewis (Director of Informatics, BDGP) talked about **'Accessing the Sequence'** and introduced the 'GeneScene' genome browser and the 'GadFly' Genome Annotation Database of *Drosophila*. GeneScene displays the transcription units of the computationally identified genes on a graphical display of the chromosomes. GadFly allows querying of the database of annotated genes on the basis of symbol, genome location, molecular function and protein domain of the encoded products.

The day after the Genome Workshop in Pittsburgh, 24 March, the 'Drosophila Genome' issue of *Science* (vol **287**, issue 5461) published the results of the sequencing and annotation collaboration, and several accompanying articles and commentary pieces. That same day, GenBank released the annotated genome sequence data and this wealth of data is now freely available through GeneScene and GadFly on FlyBase and at the BDGP, and also from the NCBI [10]. Extraordinary! Continuing a century-long tradition of community cooperation,

Copyright © 2000 John Wiley & Sons, Ltd. 

fly people are responding by offering information to improve this Release 1.0 annotation. The first update, about the 'scribbled' gene, was sent to FlyBase early in the morning of 24 March from David Bilder of Harvard University, and updates have been coming in ever since (authors' own experience).

### What's new?

What has this sequencing project given the research community that it did not have before? Before 24 March there were almost 13 000 chromosomal genes listed in FlyBase. Of these, more than 2500 have now been placed on the genome sequence and more than 11 000 'new' genes have been identified. All the 13 601 genes defined during the sequencing and annotation have been analysed computationally using sequence comparisons, and with limited human curation, for function.

The next year will see directed sequencing by the BDGP, resulting in gap closure and sequence error resolution. The annotation will be revised by FlyBase, incorporating the new sequence data, more full-length cDNA sequences from the *Drosophila* Gene Collection, the P-element insertion data produced by the BDGP in their gene disruption projects, and input from the *Drosophila* community. This re-annotation process will result in the correlation of many of the 'new' genes with previously known, but not sequenced, genetic loci, such as those defined primarily by phenotypic analysis.

### What difference will it make? A few examples . . .

Many research projects begin with a phenotypically interesting mutation caused by the insertion of a P-element transposon. A short stretch of genomic sequence to either side of the insertion site will now be sufficient to map the insertion to its location; the annotation will immediately provide a candidate gene for the function revealed by the interesting mutant phenotype. Even for chemically-induced mutations, traditionally more difficult to pin down to a molecular alteration, the sequence provides new possibilities for identifying SNP markers for recombination mapping, to narrow down target regions for full sequence analysis in mutant strains.

For anyone interested in a particular new gene (and a notable 23% of the fly's genes have no obvious homologue in another organism [1]) an obvious route into genetic analysis is to ask about its mutant phenotype. A chromosome aberration that deletes the gene can be enormously useful in getting started. 'Deficiency Kits' of deletion chromosomes which systematically cover large sections of the genome have long been available from the Bloomington *Drosophila* Stock Center [6]. The possibility of using microarray analysis to map the breakpoints of the Deficiency Kit deletion chromosomes to the transcription unit map is exciting. Additionally, new methodologies for generating custom-designed deletions are emerging. One example exploits nested P-element/hobo transposons which can be mobilized throughout the genome by P transposition, localized by sequence of the insertion site, and imprecisely excised by hobo remobilization, to create deletions, by abortive hobo transposition [11].

An allelic series of alterations can now be generated and identified for any sequenced gene, thanks to a combined chemical mutagenesis/automatic denaturing HPLC detection technique, as presented by Charles Dearolf (Massachusetts General Hospital) in the meeting's 'Technical Advances' workshop (also discussed in [3]. Other promising news in this workshop came from Yikang Rong (University of Utah), who reported success with targeted gene replacement, in this case replacing a mutant yellow gene with the wild-type copy [13].

Those newly discovered genes that represent orthologues of human disease genes will seed new projects involving the definition of a mutant phenotype, either by knock-out or by overexpression. It seems likely that these projects will spawn new collaborations between vertebrate biologists and drosophilists; the fruit fly may reach areas previously unaccustomed to its little ways, through the universality of the genetic code.

Knowing the full complement of transcription units will permit genome-wide analysis of expression patterns using microarray technology. This is clearly an area of interest for the fly world, as evidenced by the well-attended Microarray Workshop at last year's 40th Annual *Drosophila* Research Conference, Seattle, led by Ken Burtis (U.C. Davis) and this year's talk by Kevin White (Stanford University Genome Center) in the 'Technical Advances' workshop. In 'Resources in the Post-Genomic world: A community forum', Thom Kauf-

Copyright © 2000 John Wiley & Sons, Ltd.

man and Peter Cherbas (Indiana University) presented their proposal to operate a centralized service to distribute the DNA samples of the *Drosophila* Gene Collection, in conjunction with the Center for Genomics and Bioinformatics at Indiana University. Brian Oliver (NIDDK, NIH) discussed the practical issues facing experimenters considering applying microarray technology to their biological process of interest.

Proteomic analysis will permit protein–protein interactions to be probed on a scale new to *Drosophila* and, although not explicitly discussed in sessions at the Pittsburgh meeting, this technology is obviously to be anticipated. Indeed, on 21 March 2000, the BDGP and CuraGen corporation announced their collaboration to create a protein–protein interaction map of the *Drosophila* genome [7]; CuraGen has previously collaborated with Stanley Fields of the University of Washington and the Howard Hughes Medical Institute to produce a protein–protein interaction map of *Saccharomyces cerevisiae* [16].

Thus, the impact of knowing the whole genome sequence will immediately be felt at the local level, in individual research projects, where having the sequence will facilitate the progression of those projects. In addition, the emerging genomic/proteomic technologies will bring whole new classes of data to bear on our understanding of biology brought about by work on the fruit fly.

### Looking forward . . .

The paper with the charming citation, 'Genetics, Volume 1, page 1 . . .' was published in 1916 by one of the founders of *Drosophila* genetics, Calvin Bridges [2]. In this paper, Bridges described his proof that the genes, or 'heredity materials', are borne on the chromosomes. Today, only four, maybe five, scientist-generations later, we know more or less what all those genes are. The challenge, now that the problem is clearly defined, is to find out what all these genes do. To quote the angel in the *Book of Daniel* (Old Testament), '. . .and many shall run to and fro, and knowledge shall increase' (Chapter 12, Verse 4). He must have meant the people, as well as the flies . . .

### Acknowledgements

We thank Lori Wallrath and the Genetics Society of America for permission to use the splendid illustration from the conference abstract book, Francois Huet for helpful discussions, and FlyBase colleagues at Cambridge and Harvard for their memory, particularly Gillian Millburn for comments on the manuscript and Chihiro Yamada for help with scanning the picture.

### References

1. Adams MD, *et al.* 2000. The genome sequence of *Drosophila* melanogaster. *Science* **287**: 2185–2195.
2. Bridges CB. 1916. Non-disjunction as proof of the chromosome theory of heredity. *Genetics* **1**: 1–52.
3. Dearolf CR, *et al. A. Dros. Res. Conf.* **41**: 320A.
4. **http://edgp.ebi.ac.uk/**
5. **http://flybase.bio.indiana.edu/**
6. **http://flystocks.bio.indiana.edu/**
7. **http://www.curagen.com/news.html:**
8. **http://www.fruitfly.org/**
9. **http://www.hgsc.bcm.tmc.edu/**
10. **http://www.ncbi.nlm.nih.gov/PMGifs/Genomes/7227.html**
11. Huet F, *et al. A. Dros. Res. Conf.* **41**: 322C.
12. Myers EW, *et al.* 2000. A whole-genome assembly of *Drosophila*. *Science* **287**: 2196–2204.
13. Rong YS, Golic KG. 2000. *A. Dros. Res. Conf.* **41**: 84.
14. Rubin GM, *et al.* 2000. Comparative genomics of the eukaryotes. *Science* **287**: 2204–2215.
15. Rubin GM, *et al.* 2000. A *Drosophila* complementary DNA resource. *Science* **287**: 2222–2224.
16. Uetz P, *et al.* 2000. A comprehensive analysis of protein–protein interactions in *Saccharomyces cerevisiae*. *Nature* **403**: 623–627.
17. Venter JC, *et al.* 1984. Monoclonal antibodies detect the conservation of muscarinic cholinergic receptor structure from *Drosophila* to human brain and detect possible structural homology with $\alpha$1-adrenergic receptors. *Proc Natl Acad Sci U S A* **81**: 272–276.

Copyright © 2000 John Wiley & Sons, Ltd. 

